# Improving the Nulling Beamformer Using Subspace Suppression

**DOI:** 10.3389/fncom.2018.00035

**Published:** 2018-06-12

**Authors:** Kunjan D. Rana, Matti S. Hämäläinen, Lucia M. Vaina

**Affiliations:** ^1^Brain and Vision Research Laboratory, Department of Biomedical Engineering, Boston University, Boston, MA, United States; ^2^Athinoula A. Martinos Center for Biomedical Imaging, Massachusetts General Hospital, Charlestown, MA, United States; ^3^Department of Radiology, MGH, Harvard Medical School, Boston, MA, United States; ^4^Department of Neurology, MGH, Harvard Medical School, Boston, MA, United States

**Keywords:** magnetoencephalography, source localization, region of interest, beamformer, signal to noise ratio, nulling beamformer, cross-talk

## Abstract

Magnetoencephalography (MEG) captures the magnetic fields generated by neuronal current sources with sensors outside the head. In MEG analysis these current sources are estimated from the measured data to identify the locations and time courses of neural activity. Since there is no unique solution to this so-called inverse problem, multiple source estimation techniques have been developed. The nulling beamformer (NB), a modified form of the linearly constrained minimum variance (LCMV) beamformer, is specifically used in the process of inferring interregional interactions and is designed to eliminate shared signal contributions, or cross-talk, between regions of interest (ROIs) that would otherwise interfere with the connectivity analyses. The nulling beamformer applies the truncated singular value decomposition (TSVD) to remove small signal contributions from a ROI to the sensor signals. However, ROIs with strong crosstalk will have high separating power in the weaker components, which may be removed by the TSVD operation. To address this issue we propose a new method, the *nulling beamformer with subspace suppression* (NBSS). This method, controlled by a tuning parameter, reweights the singular values of the gain matrix mapping from source to sensor space such that components with high overlap are reduced. By doing so, we are able to measure signals between nearby source locations with limited cross-talk interference, allowing for reliable cortical connectivity analysis between them. In two simulations, we demonstrated that NBSS reduces cross-talk while retaining ROIs' signal power, and has higher separating power than both the minimum norm estimate (MNE) and the nulling beamformer without subspace suppression. We also showed that NBSS successfully localized the auditory M100 event-related field in primary auditory cortex, measured from a subject undergoing an auditory localizer task, and suppressed cross-talk in a nearby region in the superior temporal sulcus.

## Introduction

Magnetoencephalography (MEG) is a functional imaging modality that provides accurate temporal (~1 ms) and spatial (~1–2 cm) measures of cortical activity. MEG is extensively used in cognitive neuroscience research (Hari and Salmelin, 2012; Salti et al., 2015; Mamashli et al., 2017; Pantazis et al., 2017) and it is approved for some clinical studies, such as localization of epileptic foci (French et al., 1993; Stufflebeam et al., 2009).

In MEG, a source estimation technique is needed to translate the measured signals to estimates of the underlying neural current sources. MEG source estimation is an ill-posed inverse problem (Wang et al., 1993; Wendel et al., 2009): there is no unique solution and the solutions available are sensitive to noise. Therefore, restricting assumptions about the possible sources are needed and noise sensitivity must be mitigated by regularization. A fast, whole-brain source-localization technique is the minimum norm estimate (MNE), which is a spatially smooth estimate with a relatively wide point spread and strong crosstalk between regions of interest (ROIs) (Dale and Sereno, 1993; Hämäläinen et al., 1993; Wang et al., 1993; Hämäläinen and Ilmoniemi, 1994; Dale et al., 2000). Due to its fast computation time MNE is well-suited to conduct exploratory studies.

Another class of methods, called beamformers, have become increasingly popular for MEG and EEG source estimation, due to their goal to minimize cross-talk. The beamforming method was originally formulated for radar and sonar to pick up signals from specific transmitters while attenuating those from other locations (Van Veen and Buckley, 1988; Hillebrand et al., 2005). Beamformers have since been used in numerous applications including wireless communication, astrophysics, and biomedical signal processing (Baillet et al., 2001; Hillebrand et al., 2005). Broadly, the beamforming involves an array of sensors and combines the signal recorded by each sensor to increase the signal/noise ratio and focuses the entire sensory array on a central spatial location. In brain imaging, MEG and EEG beamformer methods are used to measure the location, magnitude and direction of the magnetic fields resulting from the electric currents flowing inside the brain (MEG) or the electric potential on the scalp surface (EEG).

Although several flavors of beamformer algorithms exist, we are specifically focusing on those algorithms that are closely related to the specific method we are proposing, the *nulling beamformer with subspace suppression* (NBSS). The Linearly Constrained Minimum Variance (LCMV) beamformer is frequently used in MEG data analysis because it is explicitly aimed at suppressing cross-talk (Van Veen and Buckley, 1988; Spencer et al., 1992; Van Veen et al., 1997; Robinson and Vrba, 1999; Hui et al., 2010). Simulations have shown that the LCMV method results in greater spatial accuracy compared to the MNE method (Hadjipapas et al., 2005). The LCMV method, like other adaptive beamformer methods, makes the assumption that the sources are uncorrelated (Reddy et al., 1987; Hillebrand et al., 2005). However, due to cortico-cortical communication which occurrs even at rest, the signals are invariably correlated across cortical regions (Reddy et al., 1987; Hillebrand et al., 2005).

Several methods have been recently developed for addressing correlated sources. For example, the dual-core beamformer specifically isolates pairs of correlated sources (Brookes et al., 2007; Diwakar et al., 2011). The nulling beamformer (NB) method introduces additional constraints on the LCMV beamformer to suppress contributions from more than two cortical sites (Hui et al., 2010). In the NB method, a predefined set of ROIs is used and it is assumed that all the sources originate from these ROIs. This assumption is suitable for hypothesis-driven studies, where the goal is to compute activity in the ROIs hypothesized to be involved in the neural implementation of a specific task.

The nulling beamformer requires an additional step to avoid overconstraining the solution: the gain matrices mapping the sources in the ROIs to sensor signals are reduced in rank through truncated singular value decomposition (TSVD), which retains a specified number of the largest singular values. However, the TSVD does not address the problem of source separation, and thus the resulting components may be strongly correlated with those of neighboring ROIs.

We propose a novel beamformer method, NBSS, which aims to modify the mapping from sensor space to source space in order to suppress high-crosstalk components. By doing so, we are able to reduce cross-talk between nearby sources for cortical connectivity analysis that has reduced contamination from signal interference. The NBSS method uses a tuning parameter to control the trade-off between the maximal amount of cross-talk and the signal power of an ROI. In two simulations we compared the performance of MNE, NB, and NBSS to measure cross-talk among predefined ROIs. The outcome of the simulations show that NBSS outperformed NB and MNE in reducing cross-talk while retaining signal power.

## Methods

In this section we will briefly define the MNE, the nulling beamformer (NB), and the NBSS method that we propose.

### Minimum norm estimate

The measured MEG signal vector y(*t*) at time *t* is assumed to be the sum of contributions of all neural sources:

(1) $$\begin{array}{r} \text{y}\left(t\right)=\sum {\text{g}}_{i}x_{i}\left(t\right)+\text{n}\left(t\right)=\text{G}\text{x}\left(t\right)+\text{n}\left(t\right), \end{array}$$

where *x*_*i*_(*t*) is the time course of the *i*th source, **x**(*t*) is the collection of time courses into a vector, **g**_*i*_ is the gain vector (column vector of the forward matrix **G**) for that source, and **n**(*t*) is additive noise. The MNE, **x**_***MNE***_(*t*), is the linear inverse estimate:

(2) $$\begin{array}{r} {\text{x}}_{MNE}\left(t\right)={\text{R}\text{G}}^{T}{\left({\text{G}\text{R}\text{G}}^{T}+\text{C}\right)}^{-1}\text{y}\left(t\right), \end{array}$$

where **R** and **C** are the source and noise covariance matrices, respectively (Dale et al., 2000).

### Nulling beamformer

The nulling beamformer (NB) method is based on the LCMV beamformer (Van Veen et al., 1997). This model assumes N distinct sources, which can be discrete current dipoles or cortical patches with uniform activity. The aim is to find a linear operator **w**_*i*_ that, when applied to **y**(*t*), yields unit gain for each source *i* while the contribution from other sources is minimized. The nulling beamformer introduces the additional constraint that attempts to eliminate the effects of all other confounding sources from the source location of interest (Hui et al., 2010). Thus, for each source, a constraint is applied in order to remove the signal contribution from other sources. The set of constraints for large number of ROIs will overconstrain the system. Therefore, the sources must be limited to a small number *P* of ROIs. To further reduce the number of constraints, a TSVD is used to obtain a rank *L* approximation for the gain matrix **G**_*p*_ for each ROI patch, indexed by *p*:

(3) $${\text{G}}_{p}^{L}={\text{U}}_{p}^{L}{\text{S}}_{p}^{L}{\left({\text{V}}_{p}^{L}\right)}^{\text{T}},$$

where ${\text{S}}_{p}^{L}$ = diag(σ_p, 1_, …, σ_p, *L*_) is a diagonal matrix of largest singular values of **G**_*p*_ and the columns of ${\text{U}}_{p}^{L}$ and ${\text{V}}_{p}^{L}$ contain the first *L* left and right singular vectors, respectively.

With the TSVD applied to each cortical ROI we implemented the nulling beamformer method as defined in (Hui et al., 2010). The following matrices for P cortical patches were constructed:

(4) $$\begin{array}{r} H=\left[{\text{U}}_{1}^{L},\ldots ,{\text{U}}_{P}^{L}\right] \end{array}$$

(5) $$\begin{array}{r} B_{p}=\left[{\text{r}}_{d}^{1}{\text{V}}_{1}^{L}{{\text{S}}_{1}^{L}}^{-1},\ldots ,{\text{r}}_{d}^{N}{\text{V}}_{P}^{L}{{\text{S}}_{P}^{L}}^{-1}\right] \end{array}$$

where ${\text{r}}_{d}^{i}=1$ for *i* **=** *p* and ${\text{r}}_{d}^{i}=0$ for *i* **≠** *p*. Using these matrices, the following weight vector was constructed for each patch p:

(6) $$\begin{array}{r} w_{p}=C^{-1}H{\left(H^{T}C^{-1}H\right)}^{-1}B_{p}^{\text{T}} \end{array}$$

where **C** is the covariance matrix of the measurement data. The nulling beamformer source timecourses **x**^NB^_*p*_(*t*) at the *p*th ROI is then:

(7) $${\text{x}}_{p}^{\text{NB}}\left(t\right)={\text{w}}_{p}^{T}\text{y}\left(t\right).$$

### TSVD of nulling beamformer may remove separable ROI activity

The nulling beamformer works best when the projections between any pair of ROI gain matrices **G**_*p*_ and **G**_*q*_ are small, that is they correspond to low cross talk. However, if this condition does not hold, such as when the distance between the ROIs is small, the nulling beamformer's nulling constraint may mutually nullify large contributions of each ROI's signal. Distant ROIs will have small magnitude components that overlap which are truncated through the TSVD.

### Using “subspace suppression” in place of TSVD

We propose a method, *subspace suppression* (*SubS)*, which will suppress singular values of the gain matrices whose associated basis vectors have high cross talk. To provide greater suppression of crosstalk we couple this method with the nulling beamformer. In the NBSS method, we first compute the projections onto a particular singular value in an ROI's gain matrix from all other basis vectors from the other ROI gain matrices. We compute for the amplitude of the projections from the other ROIs (indexed by *q*) onto the *p*th ROI's singular value's basis vectors:

(8) $$\begin{array}{r} \sigma_{q\to p,l}=\overset{K}{\underset{k=1}{\sum }}|{\text{u}}_{p,k}^{T}{\text{G}}_{q,l}{\text{v}}_{p,k}| \end{array}$$

K refers to the number of singular values in **G** while k indexes the rows in **U**_*p*_ and **V**_*p*_, which are the left and right singular value matrices of the *p*th ROI, **u**_*p, k*_ and **v**_*p, k*_ are the rows of U and V respectively, and ${\text{G}}_{q,l}={\text{U}}_{q}^{L}{\text{S}}_{q}^{l,L}{\left({\text{V}}_{q}^{L}\right)}^{\text{T}}$ where ${\text{S}}_{q}^{l,L}$ is the singular value matrix ${\text{S}}_{q}^{L}$ with all elements set to zero except for the *lth* singular value along the diagonal. The amount of crosstalk contribution from all ROIs *q* to ROI *p* is the summation of the following variables:

(9) $$\begin{array}{r} \sigma_{CT,p,l}=\overset{P}{\underset{q=1,q\neq p}{\sum }}\sigma_{q\to p,l}=\overset{P}{\underset{q=1,q\neq p}{\sum }}\overset{K}{\underset{k=1}{\sum }}|{\text{u}}_{p,k}^{T}{\text{G}}_{q,l}{\text{v}}_{p,k}| \end{array}$$

This value is normalized through dividing by the summation of cross-talk contributions among all ROIs:

(10) $$\begin{array}{r} {\hat{\sigma }}_{CT,p,l}=\frac{\sigma_{CT,p,l}}{\sum_{l=1}^{L}\sigma_{CT,p,l}} \end{array}$$

The goal of developing the NBSS method is to control the amount of cross-talk through a tuning parameter. To have a tuning parameter that does not have arbitrary limits, we placed the following design constraints on the construction of the NBSS method: the tuning parameter set to zero should not have any effect on the gain matrix, the tuning parameter set to one should completely nullify all crosstalk contributions, and the amount of suppression of a singular value should increase as the amount of cross-talk increases for a fixed value of the tuning parameter. A function that can satisfy the aforementioned properties is:

(11) $$\begin{array}{r} \sigma_{p,l}^{NBSS}=\sigma_{p,l}\frac{1-\alpha }{1+\alpha \beta } \end{array}$$

where σ_*p, l*_ is the *lth* singular value of gain matrix **G**_p_, α is the tuning parameter, and β is chosen based on the amount of suppression of the singular desired values. The β value is set such that when α is 0.5, the suppression will equal to one minus proportional amount of cross-talk in the area reflecting the amount of suppression desired at that level. The rule we defined is summarized in Equation (12):

(12) $$\begin{array}{r} \;\frac{1-\alpha }{1+\alpha \beta }=1-{\hat{\sigma }}_{CT,p,l}{|}_{\alpha =0.5} \end{array}$$

We obtain the following expression for the singular values of the new gain matrix, by solving (12) for β and inserting the β into (11):

(13) $$\begin{array}{r} \sigma_{p,l}^{NBSS}=\sigma_{p,l}\frac{1-\alpha }{1+\alpha \left(\frac{1-2{\hat{\sigma }}_{CT,p,l}}{{\hat{\sigma }}_{CT,p,l}-1}\right)} \end{array}$$

**Figure 1:**
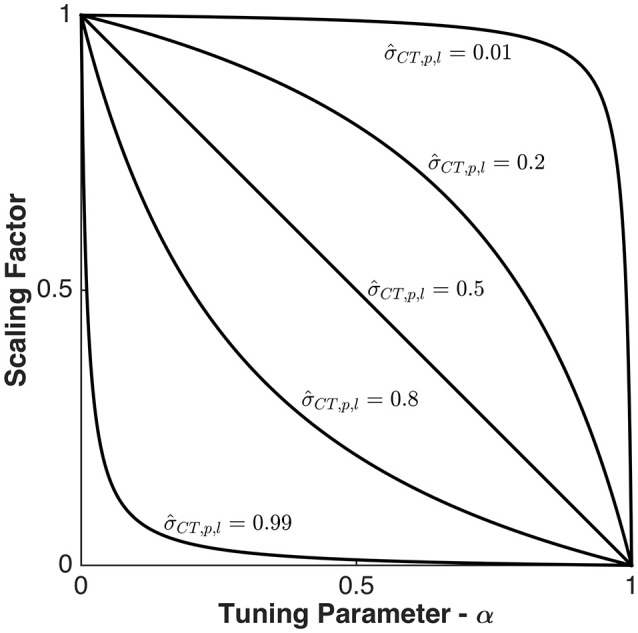
Scaling factor vs. Tuning parameter and normalized cross-talk. Scaling factor of singular values of the gain matrix of ROI p, σ_*p, l*_, as a function of the tuning parameter, α, and the normalized cross-talk contribution, ${\hat{\sigma }}_{CT,p,l}$. There is no suppression when α is 0 and complete suppression when α is 1.

As ${\hat{\sigma }}_{CT,p,l}$ increases, the amount of suppression for the same α also increases. The scaling factor α is a parameter that can range from 0 to 1, with 0 applying no cross-talk suppression, and 1 applying maximal suppression of all overlapping cross-talk components. How scaling varies as a function of α and ${\hat{\sigma }}_{CT,p,l}$ is illustrated in Figure 1 which shows the plot of the scaling factor on σ_*p, l*_ as a function of α for varying ${\hat{\sigma }}_{CT,p,l}$.

### Nulling beamformer with subspace suppression

We introduced the subspace suppression method into the nulling beamformer procedure by replacing the original singular value matrix in the TSVD in Equation (3) with the reweighted singular value matrix whose singular values are computed in Equation (13) to obtain:

(14) $$\begin{array}{r} {\text{G}}_{p}^{NBSS}={\text{U}}_{p}^{L}{\text{S}}_{p}^{NBSS}{\text{V}}_{p}^{L,T} \end{array}$$

The singular values are preweighted to minimize cross-talk between ROIs, thus allowing higher separation ability through the nulling beamformer solution without mutual signal nullification for high degrees of overlapping singular vectors.

## Simulations

In each of the simulations we defined a set of ROIs used to test two factors: the effect of various factors on the nulling constraint between two ROIs (Simulation 1), and the effect of multiple ROIs in a realistic MEG scenario (Simulation 2).

### General method for simulation of cortical signals

An MEG measurement with the 306-channel Elekta-Neuromag Vectorview system was assumed with a realistic location and alignment of the sensor array with the subject's head, obtained from an actual measurement (Vaina et al., 2010). The forward solution was computed using a single-compartment boundary-element model (Hamalainen and Sarvas, 1989). A spatial noise-covariance matrix was estimated from the MEG data in the magnetically shielded room without a subject and was used to spatially whiten both the data and the gain matrices. The noise covariance was used to add spatially colored noise to the simulated signals. We generated 1,000 samples of random Gaussian noise in the sensor space as a baseline level with no signal. The amplitude of signals from each ROI was chosen so that it would have a relative noise amplitude (RNA) of 0.1. The RNA is the ratio between the standard deviation of the noise divided by the standard deviation of the signal (Dannhauer et al., 2013). Additional 1,000 samples were generated and added to simulated activation mapped from the ROI to the sensor space.

Using the procedures detailed in the Theory section, we estimated the ROI signals from the MNE, nulling beamformer, and NBSS methods. In the computation of the MNE and beamformer methods the orientations of the sources were not constrained. The ROI signal estimate was obtained by averaging the power of the dipole amplitudes across the three dipole components and locations within each ROI. We chose the power measured from the ROI that generated the signal to be 10 times the noise level from the MNE estimate to provide a baseline of comparison between methods. For the TSVD, we vary *L* from 1 to 5.

To assess the amount of cross-talk between two ROIs, we chose a criterion often used in wireless communications to determine the quality of cross-talk suppression between channels, the signal-to-interference ratio (SIR) (Andersin et al., 1998; Jeske and Sampath, 2001). We expressed SIR as the log ratio of the power of the source at a location to the power of interfering sources at that location:

(15) $$\begin{array}{r} SIR_{p}=10\log_{10}\frac{P_{p\to p}}{\sum_{q\neq p}^{P}P_{q\to p}} \end{array}$$

where *P*_*q*→*p*_ is the power in ROI *p* due to signal generated in ROI q, which we estimate through simulations. At a level of 0, the SIR has the same power as other ROIs. We will use the SIR measure to compare the MNE, NB, and NBSS methods.

### Comparisons of the minimum norm estimate, nulling beamformer, and nulling beamformer with subspace supression methods

We applied the three methods, MNE, NB, and NBSS methods, to two simulations. In the first simulation, we tested the effects of different parameters on the SIR in the three methods. We sampled a vertex (the reference) in the intraparietal sulcus (IPS) and a random vertex (the test vertex) at a random distance away. The test vertex was allowed to be from 2 to 10 cm away at a 2 cm interval with a random jitter of 0.1 cm. For each vertex, we defined a spherical region of 1 cm radius. Using the MNE, nulling beamformer (NB), and the NBSS method we proposed, we calculated SIR between the reference vertex and the test vertices. In the NBSS method, the tuning parameter alpha varied from 0.05 to 0.95 in 0.05 steps. Subsequently, we chose the alpha parameter with maximal SIR. We repeated the simulation 100 times, randomly choosing new reference vertices within IPS. This produced 100 simulated “trials” for each sampled vertex point.

**Figure 2:**
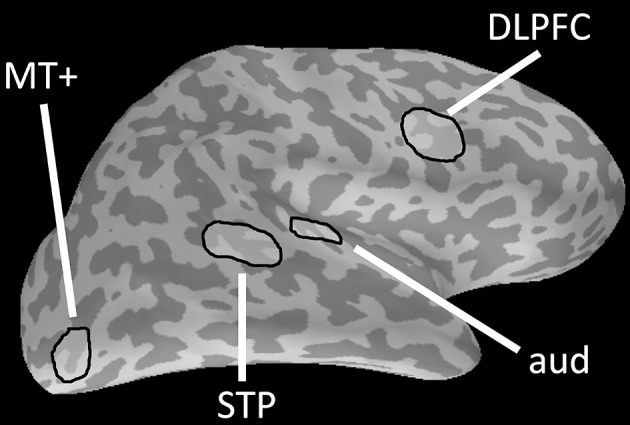
Regions of Interest on right hemisphere cortical surface. Four regions-of-interest (ROIs, green) on the right hemisphere cortical surface of a single subject (adapted from [17]): MT+, superior temporal sulcus (STP), primary auditory area (aud), and dorsolateral prefrontal cortex (DLPFC).

In the second simulation we used a set of cortical ROIs activated in one of our recent MEG study on a cognitive task on visual and auditory motion (Vaina et al., 2010). We investigated four right-hemisphere ROIs: MT+, superior temporal polysensory area (STP), primary auditory area (aud), and the dorso-lateral prefrontal cortex (DLPFC) (Figure 2). We compared the cross-talk with SIR as resulted from using the MNE, NB, and NBSS methods. We computed 1000 simulated “trials” of independent single samples generated from each of the regions.

In the (Vaina et al., 2010) study, we measured MEG data from a subject undergoing an auditory localization task. We presented a pure 300 Hz auditory tone to both ears at 69 dB for 1 s. The auditory tone induces a peak in the primary auditory cortex at 100 ms from auditory onset, labeled the M100 evoked field (Reite et al., 1978). Our interest was to localize the signal in the primary auditory area (aud) and minimize cross-talk in the neighboring area STP. Therefore, we reconstructed the signal in the primary auditory (aud) and STP regions using the MNE, NB, and NBSS methods and compared the amplitude at the M100 peak.

## Results and discussion

### Simulation 1

**Figure 3:**
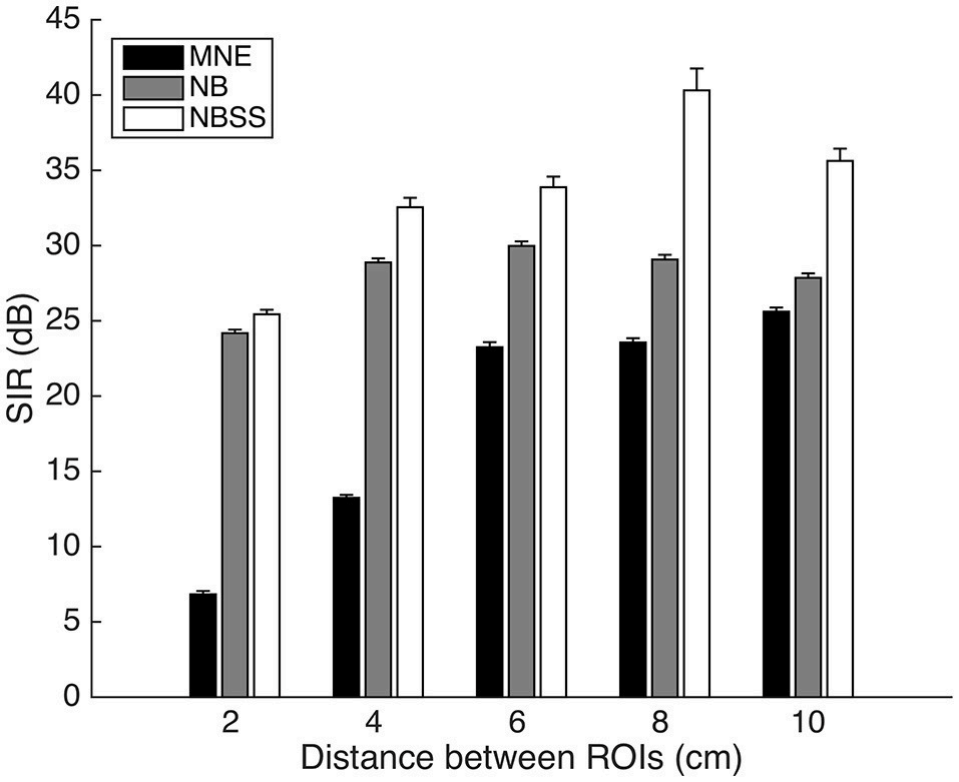
Signal to Interference Ratio (SIR) between 1 cm radius regions of varying distance. Error bars represent 1 standard error over 100 repetitions.

Our first simulation investigated how the SIR is affected by distance between two ROIs as distance between them is increased. Figure 3 shows that the nulling beamformer (NB) is significantly greater in SIR than the MNE, even for the 2 cm distance [*t*_(198)_ = 54.48, *p* < 0.001]. The NBSS provides higher SIR values at all distances [*t*-test at 2 cm between NB and NBSS, *t*_(198)_ = 3.36, *p* = 0.001].

**Figure 4:**
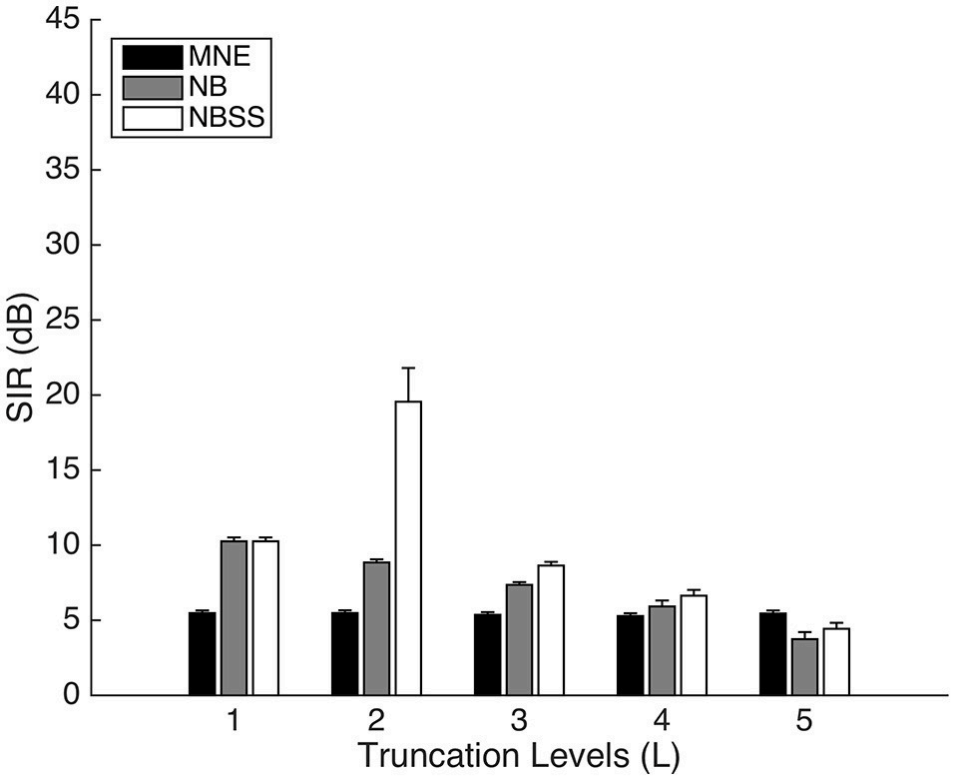
Signal to Interference Ratio (SIR) between 1 cm radius regions of varying truncation levels in NB and NBSS methods. Error bars represent 1 standard error over 100 repetitions.

For the shortest distance that we tested (2 cm), we varied the effect of the truncation amount of the TSVD for 1 through 5 dimensions (Figure 4). With L = 1, there is no difference between NB and NBSS methods, since NBSS relies on modulating the gain between components. However, in this situation, both NB and NBSS (*M* = 10.26, *SD* = 2.59) have higher SIR than MNE (*M* = 5.47, *SD* = 1.77) [*t*_(198)_ = 15.15, *p* < 0.001]. However, as the number of components increased, the effectiveness of the NB and NBSS methods decreased after L = 2. At L = 5, SIR with NBSS (*M* = 5.45, *SD* = 2.7) was lower than in MNE (*M* = 4.43, *SD* = 4.46) [*t*_(198)_ = 1.98, *p* = 0.049]. The decrease of SIR for L > 2 may be due to increased signal overlap between the two ROIs.

**Figure 5:**
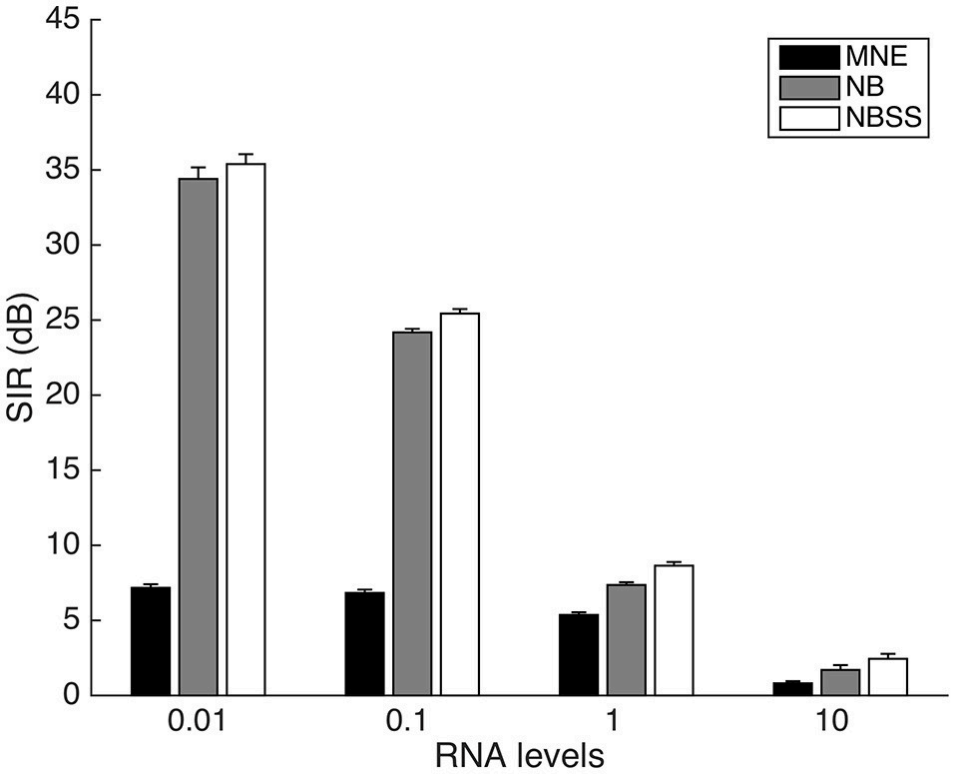
Signal to Interference Ratio (SIR) between 1 cm radius regions of varying Residual Noise Amplitudes. Error bars represent 1 standard error over 100 repetitions.

We fixed the truncation amount to L = 3 and varied the residual noise amplitude (Figure 5). The SIR difference [*t*_(198)_ = 6.12, *p* < 0.001]. However, with an RNA level of 10 where the signal is below the noise level on each trial, the NB (*M* = 1.71, *SD* = 1.39) and NBSS (*M* = 2.44, *SD* = 3.22) methods have significantly higher SIR than MNE (*M* = 0.81, *SD* = 1.40) [between NB and MNE *t*_(198)_ = 2.54, *p* = 0.012]. NBSS has slightly higher SIR than NB, but is not significant [*t*_(198)_ = 1.61, *p* = 0.110].

**Figure 6:**
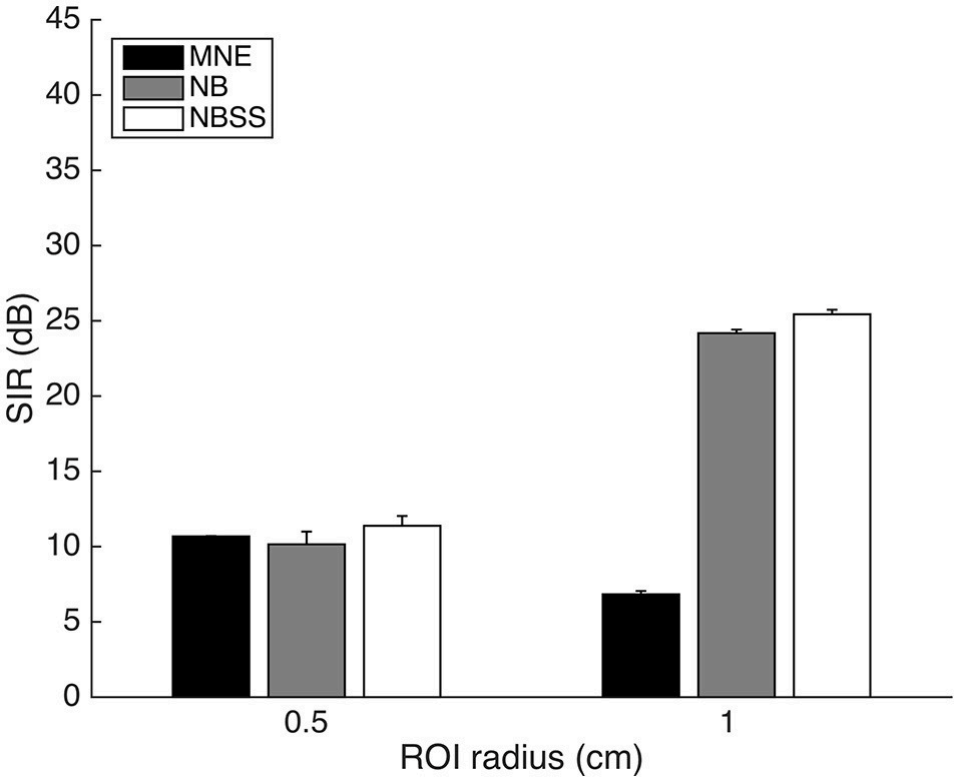
Signal to Interference Ratio (SIR) between regions of fixed 2 cm distance of 0.5 cm and 1 cm radii. Error bars represent 1 standard error over 100 repetitions.

In Figure 6, we show that the SIR is affected by the size of the ROI (Figure 6). We fixed the distance to 2 cm from the center of the ROI and compare a 0.5 cm radius to a 1 cm radius. MNE signal power dropped significantly with a larger ROI radius (radius = 0.5 cm, *M* = 10.67, *SD* = 0.22; radius = 1 cm, *M* = 6.83, *SD* = 2.17), since the ROIs were physically closer and therefore spreading more signal into the neighboring area [*t*_(198)_ = 18.20, *p* < 0.001]. However, since NB and NBSS methods selectively choose signal components that best suppress cross-talk, SIR was improved with larger radius, due to having more signal components that can be manipulated by reweighting singular values using NBSS [e.g., for NBSS at radius = 0.5 cm, *M* = 11.38, *SD* = 6.51; radius = 1 cm, *M* = 25.43, *SD* = 3.01, *t*_(198)_ = 19.64, *p* < 0.001].

### Simulation 2

**Figure 7:**
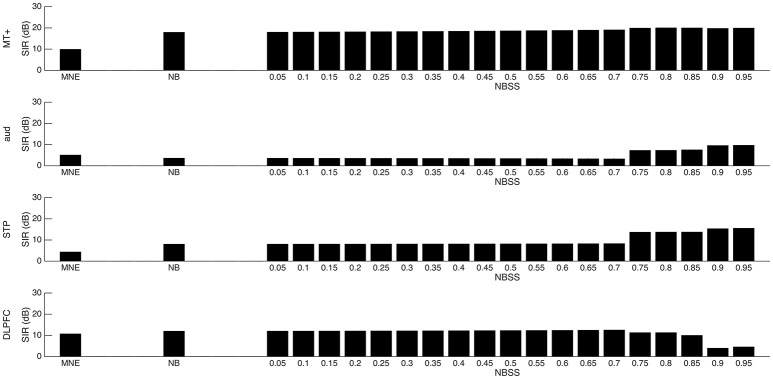
Signal to Interference Ratio (SIR) between four regions. Four regions of interest are defined in simulation 2 (MT+, aud, STP, DLPFC). SIR is measured in each region and with the minimum norm estimate (MNE), nulling beamformer (NB), and nulling beamformer with subspace suppression (NBSS) with alpha tuning parameter ranging from 0.05 to 0.95 at 0.05 intervals.

The second simulation modeled a more realistic scenario using multiple ROIs. Figure 7, shows the SIR between the MNE, NB, and NBSS methods. The NBSS method outperformed NB and MNE in all cases. The NB method, for most ROIs resulted in higher SIR than the MNE method, except when applied to the aud ROI. This may be due to strong cross-talk from STP, which is close to the aud area. As alpha varied, the SIR increased or decreased in non-continuously. The reweighting procedure in the NBSS method reorders singular values, which are ranked by their corresponding power. Therefore, the reordering drastically changed the projections between sensor and source space when varying alpha.

**Figure 8:**
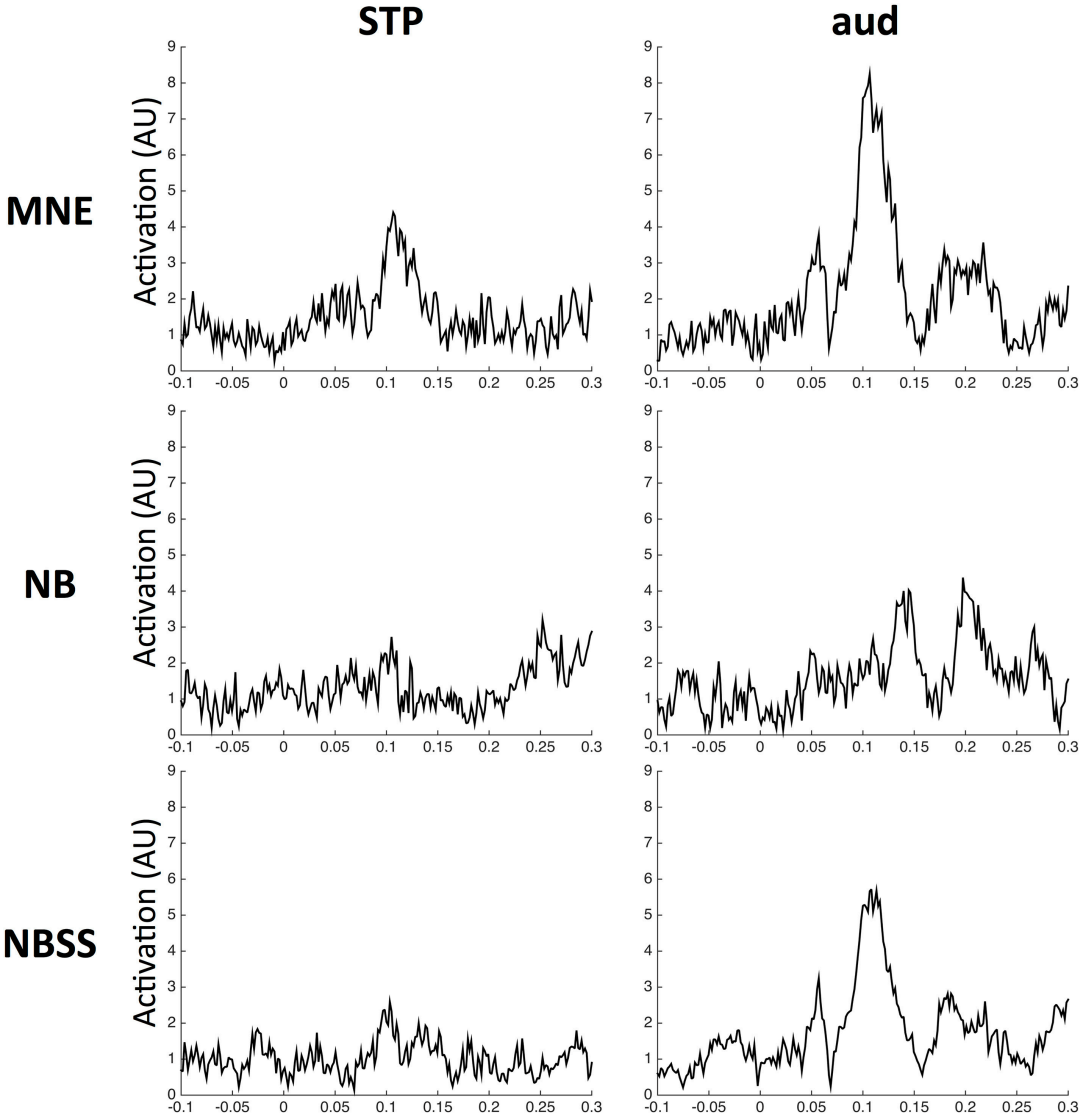
Signal Amplitude in auditory cortex and STP. Amplitude is normalized such that noise amplitude has consistent height in all figures. Time is relative to the auditory tone onset (at 0 s) therefore including a silent period (−0.1 to 0 s) and auditory tone period (0 to 0.3 s).

Using the optimal parameters showed in Figure 7, we estimated activation in the primary auditory cortex (aud) and the STP area over all trials from −0.1 to 0.3 s using each of the 3 methods using the auditory localizer MEG data from one subject in (Vaina et al., 2010). We were interested in measuring the auditory M100 peak, occurring at approximately 100 ms. The results are shown in Figure 8. Although the amplitude in aud using MNE is higher than the others (*z* = 8.55, *p* < 0.001), there is significant signal spread into STP (*z* = 4.51, *p* < 0.001). NB effectively suppresses the signal in STP (*z* = 2.78, *p* = 0.003), but also causes significant loss of signal in aud (*z* = 2.91, *p* = 0.002). NBSS recovered the high amplitude signal in aud, similar to the MNE solution (*z* = 5.96, *p* < 0.001). The amplitude of the signal in STP, however, was suppressed, but was above significance (*z* = 2.94, *p* = 0.002).

## Conclusion

The nulling beamformer effectively suppresses cross-talk between distant cortical ROIs. However, for ROIs that are close together, the preliminary step of applying the TSVD may remove components that represent the underlying signal. We showed that that the nulling beamformer significantly suppressed signal power in closely neighboring ROIs, thus allowing the measurement of cortical connectivity between closer sources with reduced interference. Importantly, the *subspace suppression method* reduces the contributions of highly overlapping gain components and thus it prevents the removal of components that significantly separate the signals between ROIs.

In the simulations described in this paper, we optimized SIR through a discrete sampling over the range of values between 0 and 1. We have also shown that while SIR provides a means of comparing signal strength to the combined cross-talk between regions, instead it may be preferred to minimize the maximal cross-talk. Through this optimization, the largest component of cross-talk would be minimized. In future work, we will formalize the optimization of the alpha parameter through SIR and explore alternative optimization measures.

The approach described in this paper is the improving of source localization during real-time applications. By estimating noise and data covariances, this method can be used to separate the signal in multiple ROIs in real-time. Wu et al. (2012) developed a near maximum-likelihood source estimation that relies on nonlinear optimization through search. However, due to the use of a parameter search, this method cannot be used in real-time application of source estimation.

## Author contributions

KR developed the proposed algorithm, carried out the simulations, and co-wrote the manuscript. MH assisted in development of the algorithm, advised on MEG principles, and assisted with writing the manuscript. LV was involved and oversaw all stages of the paper (development of algorithm, simulations, co-writing manuscript).

### Conflict of interest statement

The authors declare that the research was conducted in the absence of any commercial or financial relationships that could be construed as a potential conflict of interest.
